# Full-Length Genome Sequence of Type M/*emm83* Group A Streptococcus pyogenes Strain STAB1101, Isolated from Clustered Cases in Brittany

**DOI:** 10.1128/genomeA.01459-14

**Published:** 2015-01-22

**Authors:** Nicolas Soriano, Pascal Vincent, Gabriel Auger, Marie-Estelle Cariou, Séverine Moullec, Vincent Lagente, Jean-François Ygout, Samer Kayal, Ahmad Faili

**Affiliations:** a Laboratoire de Microbiologie et Immunologie, Facultés de Médecine et de Pharmacie, Université Rennes 1, Rennes, France; b Service de Microbiologie et Hygiène Hospitalière, CHU Pontchaillou, Rennes, France; c IGDR-UMR 6290-CNRS, Université de Rennes 1, Rennes, France; d UMR991-INSERM, Université de Rennes 1, Rennes, France; e Laboratoire de Biologie, CH de Bretagne Sud, Lorient, France

## Abstract

Here, we announce the complete annotated genome sequence of a Streptococcus pyogenes M*/emm83* strain, STAB1101, isolated from clustered cases in homeless persons in Brittany (France). The genome is composed of 1,709,790 bp, with a G+C content of 38.4% and 1,550 identified coding sequences (CDS), and it harbors a Tn*916*-like transposon.

## GENOME ANNOUNCEMENT

Streptococcus pyogenes, or group A Streptococcus (GAS), is an important Gram-positive human pathogen that causes a wide variety of infectious diseases ranging from noninvasive to life-threatening in severity (1). GAS strains are currently typed by sequencing the 5′ hypervariable region of the *emm* gene, which encodes the M protein (2). Studies of large numbers of GAS isolates worldwide have shown that only a few genotypes account for the most invasive infections (3). The *emm* genotypes that cause rare disease are mainly described during epidemics (4–7). We recently described the clonal spread of GAS M*/emm44* and sequenced a virulent strain (STAB901) isolated in 2009 (7, 8). Subsequent to the extinction of M/*emm44* GAS at the end of 2010 (our unpublished data), we observed clustered cases of infections due to M/*emm83.* This is a rare genotype that has been reported in injecting drug users (IDUs) in the United Kingdom (9). We recorded 7 new cases of infections during the summer of 2011, from which 5 patients lived in the same town, and 4 had poor living conditions (homeless and/or IDUs).

In order to understand bacterial epidemics and to add new information to the emerging field of molecular pathogenomics, we sequenced and annotated the whole genome of an M/*emm83* group A Streptococcus invasive strain (necrotizing fasciitis) isolated in Brittany, named STAB1101.

The STAB1101 strain grown in Todd-Hewitt medium supplemented with 0.2% yeast extract (THY) and DNA for sequencing was extracted and purified using the phenol-chloroform technique. Genomic DNA was sequenced using HiSeq 2000 technology (Illumina, Inc., San Diego, CA), and the paired-end library was built using the Montpellier GenomiX (MGX) facility of the CNRS in Montpellier, France. There were a total of 37,090,274 high-quality reads giving an average of 2,390-fold coverage of the genome, which was assembled *de novo* using the CLC Genomics Workbench version 6 software.

The resulting assembly consisted of 104 contigs, which were oriented and connected with the module Microbial Genome Finishing Tools. After reassembling, 9 gaps persisted. The gaps were filled by PCR, followed by Sanger sequencing. Genome annotation was performed in parallel using the RAST server (10) and NCBI-PGAP (http://ncbi.nlm.nih.gov/genome/annotation_prok). Prophages were identified using the PHAge Search Tool (PHAST) (11). Finally, strain STAB1101 was found to harbor a single circular genome of 1,709,790 bp, with a G+C content of 38.4%. We identified 1,550 coding sequences (CDSs), 67 tRNA genes, 18 rRNA genes, and one incomplete prophage (33 Kb, 44 CDSs, and a G+C content of 35.24%). The superantigen genes identified were *speG, speL, speM*, and *smeZ*. In addition, and as found within the chromosome of the GAS strain STAB901, the genome analysis of strain STAB1101 showed the presence of a Tn*916*-like transposon that has a close similarity to Tn*6253* (8) but does not harbor a group II intron. Its insertion site is located in a noncoding sequence between inosine 5-monophosphate and a sugar transporter.

This complete genome of a GAS strain isolated from clustered cases brings the opportunity to study its clonal origin and rate of genome diversification over the short epidemic period.

### Nucleotide sequence accession number.

The complete whole-genome sequence of S. pyogenes strain STAB1101 has been deposited in the NCBI under the accession no. CP007240.1. The version described in this paper is the first version.
